# Homes Abroad for Nurses

**Published:** 1888-03-17

**Authors:** 


### HOMES ABROAD FOR NURSES.

"One of Several who can Speak from Experience" writes from Brighton:

"I presume that Miss Wilson is not aware of the change of superintendents at the Nice Institution, or she certainly would not recommend Nurse Johnson to go there. Formerly it was a very comfortable home, but I am sorry to say all now is changed."

